# Hypereosinophilic syndrome with central nervous system involvement: a case report

**DOI:** 10.3389/fnimg.2026.1783329

**Published:** 2026-04-08

**Authors:** Wenjuan Xu, Chao Zhang, Fan Wang, Xiaomin Liu, Xiao Zhang, Fei Mao, Xinyi Wang, Xiaoyu Zhang

**Affiliations:** 1 Department of General Practice, The First Affiliated Hospital of Shandong First Medical University & Shandong Provincial Qianfoshan Hospital, Jinan, Shandong, China; 2 Department of Radiology, The First Affiliated Hospital of Shandong First Medical University & Shandong Provincial Qianfoshan Hospital, Jinan, Shandong, China; 3 Department of Orthopedic Surgery, The First Affiliated Hospital of Shandong First Medical University & Shandong Provincial Qianfoshan Hospital, Jinan, Shandong, China; 4 Department of Neurology, The First Affiliated Hospital of Shandong First Medical University & Shandong Provincial Qianfoshan Hospital, Jinan, Shandong, China

**Keywords:** case report, cerebral infarction, hypereosinophilic syndromes, intracranial artery stenosis, vascular wall enhancement, vasculitis

## Abstract

Cerebral infarction is the most common neurological complication in patients with hypereosinophilic syndromes (HES), typically occurring in border-zone regions. However, intracranial artery stenosis is rarely observed in HES, and the underlying mechanisms of cerebral infarction remain largely unknown. Here, we report a case of HES complicated by acute ischemic stroke secondary to severe stenosis of left middle cerebral artery (MCA). A diagnosis of idiopathic HES was established based on eosinophilia (14.08%) in bone marrow aspiration and negative genetic testing. Without contraindications, intravenous thrombolysis with alteplase was administered, resulting in a decrease of the National Institutes of Health Stroke Scale score from 13 to 2. High-resolution magnetic resonance imaging (HR-MRI) showed homogeneous, concentric wall thickening and enhancement in the terminal segments of the left internal carotid artery and at the origin of the MCA, indicating an inflammatory process. Follow-up HR-MRI at 17 months demonstrated a reduction in vessel wall enhancement after immunosuppressive therapy. Over the two-year follow-up period, the eosinophil count remained within the range of 0.22–1.09 × 10^9^/L, and no stroke recurrence was observed. In the literature review, only three cases of stroke associated with HES reported intracranial stenosis, all located in the M1 segment of the MCA. Their clinical outcomes improved following immunosuppressive therapy. Thus, intracranial large artery stenosis is a rare etiology of stroke in patients with HES. Homogeneous vessel wall enhancement on HR-MRI suggests an underlying vasculitis, which appears responsive to immunosuppressive therapy.

## Introduction

The hypereosinophilic syndromes (HES) are a diverse group of rare disorders defined by persistent peripheral blood eosinophilia (≥1.5 × 10^9^/L) and subsequent eosinophil-mediated organ damage. These syndromes are classified into primary neoplastic, secondary or reactive, and idiopathic subtypes (Ono et al., 2021; Ogbogu et al., 2009; Cioclu et al., 2022). Clinical manifestations are heterogeneous, most commonly involving the skin, followed by the lungs, gastrointestinal tract, heart, and nervous system. Among neurological complications, encephalopathy, sensory polyneuropathy, and cerebral infarction are the most frequent. It has been reported that approximately 12% of HES patients develop cerebral infarction, typically located in border-zone regions (Miethe et al., 2022). However, the exact mechanisms underlying cerebral infarction in patient with HES remain largely unknown.

Although several mechanisms, such as eosinophil-induced endothelial injury and thrombotic microangiopathy, a prothrombotic and hypercoagulable state, and cardiac microembolism have been proposed to explain these infarctions, the pathophysiology underlying intracranial large artery involvement in HES remains largely unexplored. Such involvement is exceedingly rare and, when present, is often associated with watershed infarction.

Here, we present a case of HES complicated by acute ischemic stroke attributable to left middle cerebral artery (MCA) stenosis. High-resolution magnetic resonance imaging (HR-MRI) revealed homogeneous, concentric wall thickening and enhancement in the terminal segments of the left internal carotid artery (ICA) and origin of the MCA, suggestive of an underlying inflammatory process. Moreover, follow-up HR-MRI demonstrated a decrease in this enhancement following immunosuppressive therapy, raising the possibility that the intracranial large artery involvement in this case was secondary to idiopathic eosinophilic vasculitis.

## Case report

A 32-year-old man presented to the emergency room on June 8, 2023, with 1 h of aphasia and right-sided limb weakness. He had a 10-year history of hypereosinophilia and thromboangiitis obliterans and was managed with cilostazol. His vascular risk factors included a 10-year smoking history and occasional alcohol consumption. He had no history of parasitic infection, allergic diseases and asthma. No history of fever, skin rash, joint pains, weight loss, or night sweats was noted. Neurological examination revealed mixed aphasia, with right upper limb muscle strength assessed as grade 0 and right lower limb as grade 2. The National Institutes of Health Stroke Scale (NIHSS) score was 13, and the Modified Rankin Scale (mRS) score was 2. Acute cerebral hemorrhage was ruled out by brain computed tomography (CT), leading to a diagnosis of acute ischemic stroke. A complete blood count showed an elevated eosinophil count (1.552 × 10^9^/L) and percentage (12.6%). Notably, the right limb strength improved to grade 4 within half an hour, and the NIHSS score decreased to 2. In the absence of contraindications, intravenous thrombolysis with alteplase (0.6 mg/kg) was administered, with a door-to-needle time of 53 min. Post-thrombolysis, the patient exhibited with only mild aphasia and right limb weakness. His NIHSS score was 2, and the mRS score was 1.

To investigate the etiology of the HES, the following tests were performed and yielded normal results: autoimmune serology (including rheumatoid factors, antinuclear antibodies, antineutrophil cytoplasmic antibody, and antiphospholipid antibody), antithrombin, protein C, protein S, thyroid function, and infection-related markers. Four months prior, a bone marrow aspiration had been performed, which revealed eosinophilia (14.08%). Genetic testing for mutations associated with myeloproliferative neoplasms and myelodysplastic syndromes, including *BCR-ABL*, *JAK2*, *CALR*, *MPL*, *RUNX1 (AML1)*, *KMT2A (MLL)*, *ETV6 (TEL)*, *PDGFR*, and *FGFR1*, was negative. A detailed drug history was obtained to exclude the effect of specific medications on peripheral eosinophilia, including allopurinol, phenytoin and carbamazepine. Based on these findings, a diagnosis of idiopathic HES was considered.

**Figure 1:**
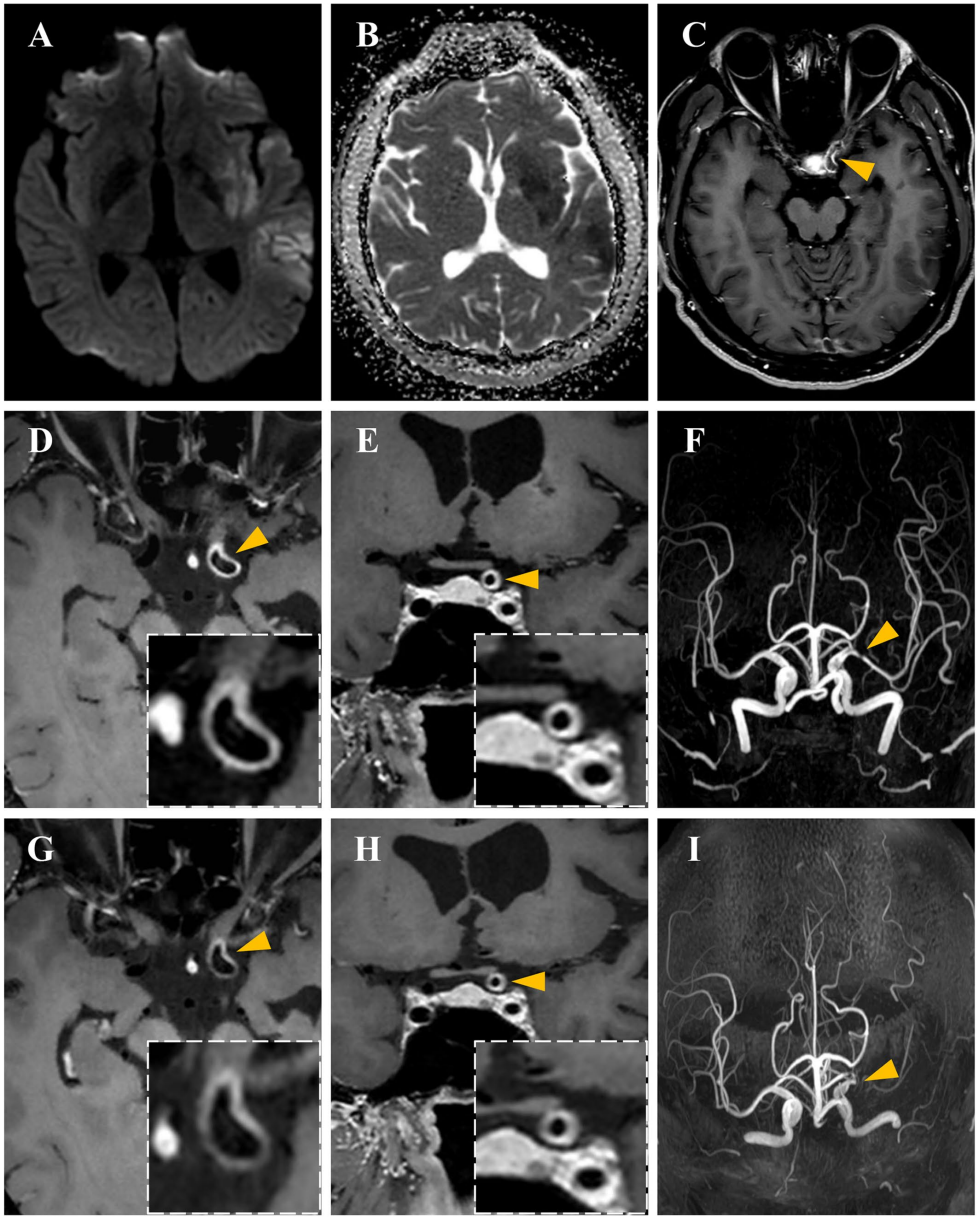
Neuroimaging features of patients with hypereosinophilic syndromes and stroke. **(A,B)** Acute ischemic infarcts in the left temporal lobe, insular lobe, and basal ganglia, showing hyperintensity on DWI and hypointensity on ADC. **(C)** Prominent enhancement of the left internal carotid artery (ICA) in contrast-enhanced MRI. **(D,E)** Homogeneous, concentric wall thickening and enhancement of the left ICA on high-resolution MRI (HR-MRI). **(F)** Severe stenosis of the M1 segment of the left MCA on magnetic resonance angiography (MRA) at admission. **(G,H)** Decreased concentric vessel wall enhancement of the left ICA on follow-up HR-MRI at 17 months; **(I)** Occlusion of the left MCA on follow-up MRA.

Brain magnetic resonance imaging (MRI) revealed multiple acute ischemic lesions in the left MCA territory (Figure 1). Magnetic resonance angiography (MRA) demonstrated severe stenosis of the M1 segment of the left MCA. Contrast-enhanced MRI showed more prominent enhancement of the left ICA compared to the right. HR-MRI was performed to detect the vessel wall lesions and showed homogeneous, concentric wall thickening and enhancement in the terminal segments of the left ICA and origin of MCA. Echocardiography and 24-h Holter monitoring showed no significant abnormalities. Doppler ultrasound of the lower extremities indicated an occlusion in the mid-to-distal segment of the left superficial femoral artery. Based on these findings, a diagnosis of idiopathic eosinophilic vasculitis involving the intracranial large arteries was considered. The differential diagnosis includes eosinophilic granulomatosis with poliangiitis (EGPA) and primary central nervous system vasculitis (PCNSV). The patient did not meet the diagnostic criteria for EGPA due to the absence of asthma and paranasal sinus involvement. The likelihood of PCNSV was also reduced, given the lack of parenchymal or leptomeningeal enhancement and the absence of steno-occlusive vascular changes or beading in multiple vascular territories.

According to the diagnosis, immunosuppressive treatment with prednisone (30 mg/day) was initiated. The patient was also treated with aspirin, clopidogrel and statin according to the guideline for acute ischemic stroke. The patient exhibited with only mild aphasia at discharge. His NIHSS score was 1, and the mRS score was 1.

Following discharge, the patient was maintained on oral prednisone (initiated at 30 mg daily with a tapering regimen of 5 mg reduction every 10 days until a maintenance dose of 10 mg daily), aspirin, and statin for secondary stroke prevention. Follow-up HR-MRI at 17 months demonstrated a reduction in concentric vessel wall enhancement in the terminal segments of the ICA and origin of MCA. However, stenosis of the left MCA progressed to occlusion. Over the two-year follow-up period, the eosinophil count remained within the range of 0.22–1.09 × 10^9^/L, and no stroke recurrence was observed.

## Discussion

The primary neurological manifestations in patients with HES include encephalopathy, peripheral neuropathy, and cerebral infarction. It is estimated that cerebral infarction occurs in approximately 12% of HES patients (Singla et al., 2024). Notably, a border-zone pattern of cerebral infarction is the most common presentation, accounting for about 57–65.6% of all stroke cases in this population (Ono et al., 2021; Miethe et al., 2022). The underlying mechanisms of stroke in HES have not yet been fully elucidated. Several hypotheses have been proposed, including eosinophil-induced endothelial injury and thrombotic microangiopathy, a prothrombotic and hypercoagulable state, and cardiac microembolism (Ono et al., 2021). In a stroke patient with idiopathic HES, pathological examination revealed thrombotic microangiopathy in the brain, evidenced by fibrin thrombi occluding the lumina of several vessels (Singla et al., 2024). Moreover, eosinophils may release cationic proteins, basic proteins, neurotoxins, leukotrienes, and prostaglandins, which can contribute to a hypercoagulable state (Wang et al., 2023). These conditions may lower perfusion pressure and alter cerebral arterial blood flow dynamics, reducing the clearance (washout) of emboli and resulting in border-zone infarctions. Additionally, elevated blood cell counts may lead to hyperviscosity, which can impair microcirculation and further diminish microemboli clearance.

Meanwhile, cardiac microembolism represents another important etiology of cerebral infarction in HES patients, often due to Löffler endomyocarditis and endomyocardial fibrosis. It was reported in a review that cardiac HES accounted for 45% of stroke cases in HES patients, commonly manifested by mural thrombi, endomyocardial fibrosis, and a restrictive pattern of heart failure (Ono et al., 2021). Pathological studies have shown thrombi entangled within ventricular trabecular muscles and along the endocardial surface, which are considered potential sources of cardiogenic cerebral embolism (Singla et al., 2024). This mechanism is further supported by transcranial doppler ultrasound studies that have detected microembolic signals in HES patients with stroke (Aida et al., 2013). Therefore, cardiac investigations is essential in patients with stroke and HES.

Involvement of the intracranial large arteries is rare in patients with HES. To our knowledge, only three cases of HES with intracranial stenosis have been reported to date, as summarized in Table 1 (Takeuchi et al., 2010; Li et al., 2020; Raut et al., 2017). In all three previously reported cases, stenosis was located in the M1 segment of the MCA, which is consistent with the findings in our patient. Among these cases, two patients presented with unilateral stenosis and one with bilateral stenosis. Follow-up intracranial vascular imaging was performed in only one of these patients, showing progression of bilateral stenosis to occlusion. Similar progression of intracranial artery stenosis was observed in our patient. The underlying mechanisms of intracranial large artery lesions in HES remain largely unknown. In our case, HR-MRI revealed homogeneous, concentric wall thickening and enhancement, a finding of intracranial vasculitis that has not been previously documented in HES patients. This is consistent with a previously reported case that described bilateral MCA wall thickening and enhancement on contrast-enhanced coronal MRI (Li et al., 2020). Vessel wall enhancement on HR-MRI is generally attributed to inflammatory processes, increased blood–brain barrier permeability, or neovascularization (Mandell et al., 2017). In another eosinophilia-related disease, EGPA, it had been identified that vasculitis typically affects both small and medium-sized blood vessels. Therefore, we hypothesized that inflammatory mechanisms may also contribute to intracranial arterial pathology in HES. The reduction in vessel wall enhancement following immunosuppressive therapy observed in our patient further supports this inflammatory hypothesis. Unfortunately, no pathological evidence is available regarding intracranial large arteries in HES. Future studies should investigate both the presence of eosinophilic infiltration within intracranial artery walls in HES and its relationship with imaging patterns of ischemic stroke. It should be noted that concentric wall thickening and enhancement can also be detected in patients with Moyamoya disease (MMD) (Kathuveetil et al., 2020). A key differentiating imaging feature of MMD is negative remodeling of the distal ICA, attributable to histopathological changes including reduction of smooth muscle cells, thinning of the media, and constriction of the internal elastic lamina. In our patient, follow-up HR-MRI at 17 months revealed no negative remodeling in the distal ICA and no vessel wall enhancement in the collateral ICA or MCA, which collectively argue against a diagnosis of MMD.

**Table 1:** Literature review of intracranial artery stenosis in patients with hypereosinophilic syndromes and stroke.

Patients	Patient 1	Patient 2	Patient 3
Author (date)	Takeuchi et al. (2010)	Raut et al. (2017)	Li et al. (2020)
Age (years)	23	53	55
Sex	Female	Male	Female
Medical history	No	Dyslipidemia, stroke	No
Initial presentations	Aphasia and right-sided hemiparesis	Slurring of speech and left sided weakness	Right limbs weakness and slurred speech
Eosinophil (/μL)	168,000	3,200	5,290
Site of stroke	Left insular cortex	Right corpus striatum	Multiple infarctions of bilateral hemisphere
Location of stenosis	M1 segment of left MCA	M1 segment of right MCA	Bilateral MCA
Bone marrow aspiration	Eosinophilia	Eosinophilia	Eosinophilia (15%)
Genetic testing	No	Negative	Negative
Treatments	Transarterial thrombolysis, anticoagulation, aspirin, prednisolone, hydroxyurea, imatinib methylate	Steroids	Vascular stent, methylprednisolone
Clinical outcome	Improved	Improved	Improved
Follow-up eosinophil (/μL)	240	32	70

For patients with idiopathic HES, corticosteroids are currently recommended as first-line therapy. In symptomatic patients who are nonresponsive to steroids, agents such as interferon-alpha, pegylated interferon-alpha (peg-IFN-α), ciclosporin, hydroxycarbamide, imatinib, and mepolizumab should be considered. Data on reperfusion therapy for acute ischemic stroke in HES patients remain limited. Thrombolysis may be beneficial if administered within the appropriate time window, as it may theoretically reverse the hypercoagulable state. In studies of PCNSV, aspirin therapy was found to be positively associated with long-term remission, an effect that may be attributed to its combined antithrombotic and anti-inflammatory properties (Pascarella et al., 2023). Similarly, in patients with antineutrophil cytoplasmic antibody-associated vasculitis (AAV), statin use has been reported to be associated with a lower incidence of relapse, possibly due to its anti-inflammatory and immunomodulatory effects (Yamaguchi et al., 2020). Aspirin and statin therapy may be reasonable considerations for acute stroke patients with HES, while their potential benefits warrant investigation in future cohort studies. The long-term prognosis of endovascular treatment remains uncertain, as inflammatory processes may cause pre-existing stenosis to progress to occlusion.

The main limitation of this study is the absence of histopathological confirmation of intracranial vasculitis, which remains the diagnostic gold standard. However, endovascular biopsy of the intracranial large arteries in our case may clinically inappropriate due to the prohibitive risk of irreversible neurological damage. In the absence of a tissue diagnosis, we utilized HR-MRI as a surrogate. HR-MRI is a well-established, non-invasive modality capable of characterizing vessel wall lesions, and the observed concentric wall thickening and enhancement followed by radiological improvement after immunosuppressive therapy strongly support the diagnosis of underlying vasculitis, effectively distinguishing it from other intracranial vasculopathies.

## Conclusion

Intracranial large artery stenosis is a rare etiology of stroke in patients with HES. Homogeneous vascular wall enhancement on HR-MRI suggests an underlying vasculitis, which appears responsive to immunosuppressive therapy.

## Data Availability

The original contributions presented in the study are included in the article/supplementary material, further inquiries can be directed to the corresponding author.
